# Design and Synthesis of *N*^1^,*N*^5^-bis[4-(5-Alkyl-1,2,4-oxadiazol-3-yl)phenyl]glutaramides as Potential Antifungal Prodrugs

**DOI:** 10.3390/molecules180911250

**Published:** 2013-09-12

**Authors:** Nageswara Rao Kode, Jean J. Vanden Eynde, Annie Mayence, Guangdi Wang, Tien L. Huang

**Affiliations:** 1 College of Pharmacy, Xavier University of Louisiana, New Orleans, LA 70125, USA; E-Mails: kode_rao@msn.com (N.R.K.); jean-jacques.vandeneynde@umons.ac.be (J.J.V.E.); annie.mayence@condorcet.be (A.M.); 2 Laboratory of Organic Chemistry, University of Mons-UMONS, Mons B-7000, Belgium; 3 Department of Chemistry, Xavier University of Louisiana, New Orleans, LA 70125, USA; E-Mail: gwang@xula.edu

**Keywords:** prodrugs, amidoximes, oxadiazoles, *Pneumocystis carinii*, *Trypanosoma brucei*

## Abstract

A facile three step synthesis of a group of *N*^1^,*N*^5^-bis[4-(5-alkyl-1,2,4-oxadiazol-3-yl)phenyl]glutaramides, *N*^1^,*N*^5^-bis[4-(1,2,4-oxadiazol-3-yl)phenyl]glutaramide and *N*^1^,*N*^5^-bis[4-(5-oxo-4,5-dihydro-1,2,4-oxadiazol-3-yl)phenyl]glutaramide is described. These products are designed to function as masked bis-amidine prodrugs of a promising *N*^1^,*N*^5^-bis[4-(N'-(carbamimidoyl)phenyl]glutaramide antifungal lead.

## 1. Introduction

**Figure 1:**
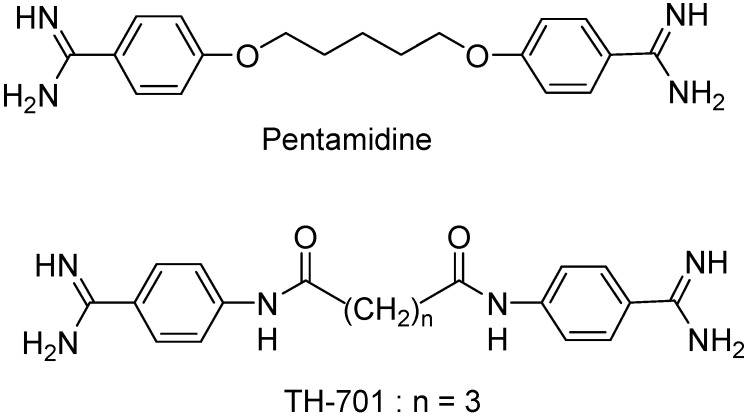
Chemical structures of pentamidine and lead compound.

We have previously reported on the synthesis of a series of alkanediamide-linked bis-benzamidines (Figure 1) and their potent *in vitro* activity against *Pneumocystis carinii*, an opportunistic fungus that causes pneumonia in immunocompromised patients, and *Trypanosoma brucei*, the parasitic protozoa that causes trypanosomiasis [1,2]. In addition to high potency, several of the tested compounds demonstrated very low cytotoxicity in the A549 human lung carcinoma cell line. From this study, bis-benzamidines linked with a pentanediamide (TH-701, Figure 1) or a hexanediamide moiety exhibited the highest selectivity indexes (defined as the ratio of the cytotoxic mammalian IC_50_ to the *P. carinii* or *T. brucei* IC_50_ values). A high selectivity index generally indicates that a compound has reasonable selectivity for binding to the disease-relevant pathogen over mammalian cells *in vitro* [3]. For example, the lead compound, *N*^1^,*N*^5^-bis[4-(N'-(carbamimidoyl)phenyl]glutaramide (TH-701, Figure 1) showed selectivity indexes of 758,667 and 252,889 against *P. carinii* and *T. brucei* respectively, whereas the selectivity indexes for the reference drug pentamidine were 48 and 12,000, respectively [2]. Further evaluation of several bis-benzamidines in an animal model of pneumocystosis indicated that *N*^1^,*N*^5^-bis[4-(N'-(carbamimidoyl)phenyl]glutaramide emerged as the most promising anti-*Pneumocystis* lead [4]. However, despite its *in vivo* efficacy and low cytotoxicity, this compound has low oral bioavailability because of the highly basic and dicationic nature of the bis-amidine functional groups. The calculated partition coefficient (cLog*P*) and pKa values for TH-701 are −0.26 and 10.99 respectively.

To overcome this drawback of low oral bioavailability in the lead compound TH-701, we decided to mask the the bis-amidine groups with prodrug functions that are expected to be more lipophilic and have lower pKa’s, thereby increasing uptake in the gastrointestinal tract. The prodrug functions that we selected are amidoximes, acylamidoximes and oxadiazoles. These prodrug groups are expected to be bioactivated by mammalian hepatic enzymes into the active amidine groups. For example, amidoximes have been shown to function effectively as prodrugs to improve the oral bioavailabity of amidine-containing drugs [5,6,7,8]. Amidoximes can be reduced to amidines via the cleavage of the N-O bond by the newly discovered mitochondrial Amidoxime Reducing Component (mARC) [9,10]. The oxadiazoles (**5a**–**g**, **6**, **7**) are designed based on the observation by Kitamura [11] that the 1,2,4-oxadiazole ring may function as a masked amidine group. Oxadiazoles in general have been reported to possess a wide range of biological activities. Some of them that are mentioned here include anti-rhino viral [12], muscarinic [13], analgesic [14], anti-inflammatory [15], anti-HIV [16] and anti-cancer [17,18].

## 2. Results and Discussion

**Figure 2:**
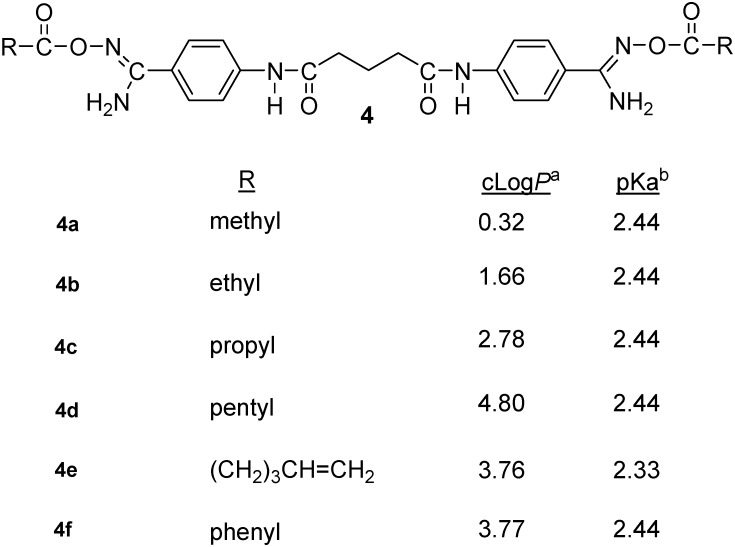
Structures of acylamidoximes **4a**–**f**.

^a^ see ref [19]; ^b^ see ref [20].

The acylamidoximes **4a**–**f** (Figure 2) may be viewed as double prodrugs since they are expected to undergo *in vivo* hydrolysis by esterases followed by reduction by mARC. The acylamidoximes are designed to increase the lipophilicity of these molecules and they may be viewed as the acyclic analogs of the corresponding oxadiazole ring-bearing compounds. The calculated partition coefficients (cLog*P*) and predicted pKa values of these compounds are shown in Figure 2.

**Scheme 1:**
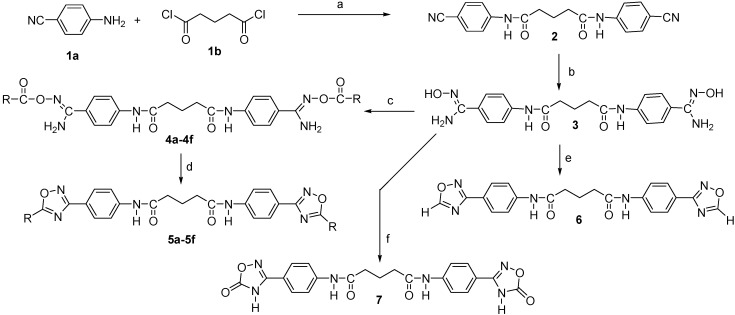
Synthetic pathways for compounds **4a**–**f**, **5a**–**f**, **6** and **7**.

**Figure 3:**
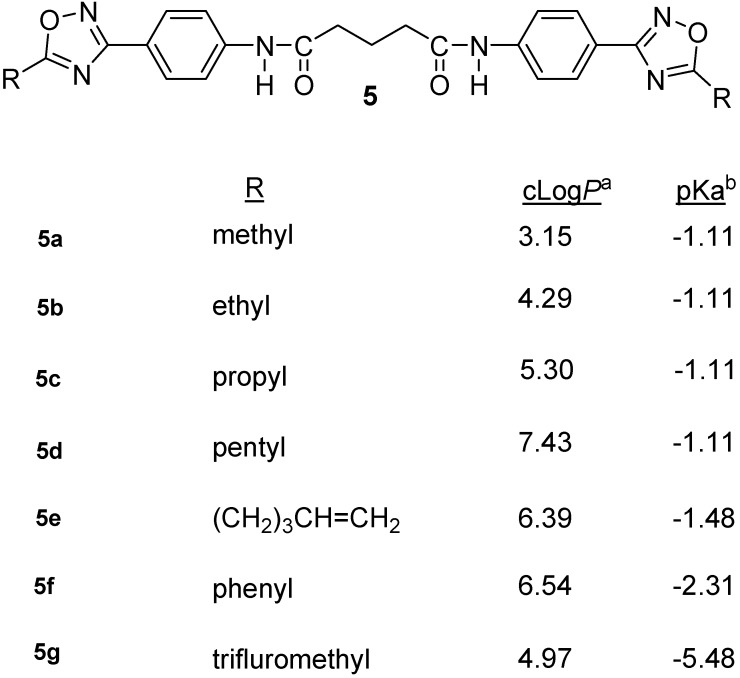
Structures of 1,2,4-oxadiazoles, **5a**–**g**.

^a^ see ref [19]; ^b^ see ref [20].

These compounds were designed based on the observation that the bis-*O*-acetylamidoxime derivative of the bis-alkylamidine, 1,12-bis(N,N’acetamidinyl)dodecane, was effective as an antimalarial prodrug when administered by the oral route [21]. At our laboratory, the isolable acylamidoximes **4a**–**f** were synthesized in high yields by the reaction of *N*^1^,*N*^5^-bis{4-[(N'-hydroxy-carbamimidoyl)pheny]}glutaramide (**3**) with the appropriate aliphatic or aromatic anhydrides in DMSO medium in the presence of triethylamine at room temperature for 18–48 h (Scheme 1). Esterification of **3** employing the appropriate acid chlorides and potassium carbonate was found to be more complex by TLC when compared to the anhydride route. The anhydride route worked very well for our molecules, often resulting in only one major product. Further, monitoring the esterification process of **3** with anhydrides by IR was very helpful where the dominant ester carbonyl absorption around 1,700 cm^−1^ could be easily recognized from the isolated crude reaction products free from the starting anhydrides. Furthermore, IR was also helpful to monitor the disappearance of the ester carbonyl and amino group absorptions of **4a**–**f** during the cyclization step leading to the formation of 1,2,4-oxadiazoles **5a**–**f** (Figure 3).

*Reagents and conditions*: (**a**) DMF, r.t.,12–24 h; (**b**) Hydroxylamine, DMSO, 70 °C, 17 h; (**c**) Appropriate anhydride, DMSO, triethylamine, r.t., 18–48 h; (**d**) DMSO, 80–90 °C, 18–24 h; (**e**) Trimethylorthoformate, boron trifluoride etherate (3 drops), 80–82 °C, 2 h; (**f**) DMSO, 1,1'-carbonyldiimidazole (CDI), 100 °C, 2 h.

Several studies suggested that the oxadiazolone ring may serve as a masked amidino group with reduced basicity, increased lipophilicity and hence better oral bioavailability [11,21,22,23,24]. The oxadiazolone derivative of an GP IIB/IIIa antagonist was shown to be metabolized into the amidine derivative using guinea pig liver homogenate and was active *in vivo* as a platelet aggregation inhibitor when administered orally in guinea pigs [11]. The oxadiazolone ring in an angiotensin II receptor antagonist was reported to increase the lipophilicity and hence oral bioavailability of the antagonist [22,23]. In addition, the oxadiazolone ring in the angiotensin II antagonist was biotransformed into an amidino group in one of the metabolites [11]. The bis-oxadiazolone derivatives of a bis-alkylamidine series were shown to be effective prodrugs that displayed oral antimalarial activity [21,24]. These observations prompted us to design and synthesize a series of bis-oxadiazoles **5a**–**g**, **6** and **7** as potential masked amidino equivalents of the parent lead compound TH-701.

General synthesis oxadiazoles involve the transformation of an amidoxime into an *O*-acyl- amidoxime by the reaction of an acid chloride [25], anhydride [26], ester [27] or an orthoester [28]. The *O*-acylamidoxime undergoes cyclization under the influence of a strong base [29] or by heating to 85 °C in 2-methoxyethyl ether [30] or by heating the sample slightly above its melting point [31]. Microwave-assisted synthesis of 1,2,4-oxadiazoles was reported [32,33], while alumina supported ammonium fluoride was reported to be a useful reagent for the reaction of amidoximes with acylchlorides under microwave solvent free synthesis [34]. Bora *et al*. [35] reported the 1,2,4-oxadiazole synthesis by the reaction of amidoximes with acid chlorides in refluxing toluene medium in presence of molecular sieves, while *O*-acetylated amidoximes, upon heating in acetic acid, yielded the 1,2,4-oxadiazoles [36]. The oxadiazolones are conveniently prepared by cyclizing the amidoxime with 1,1'-carbonyldiimidazole in 1,4-dioxane [11] or with methylchloroformate in chloroform [21] or ethylchloroformate in xylene [24]. At our laboratory, the target 1,2,4-oxadiazoles (Figure 3) were synthesized by the facile cyclization of the *in situ* generated *O*-acylamidoximes in one step by heating **3** with the appropriate anhydrides in DMSO medium at 80–90 °C, 18–24 h. (Scheme 1**)**. The calculated partition coefficients (cLog*P*) and predicted pKa values of these compounds are shown in Figure 3.

It is clear that the prodrugs **4a**–**f**, **5a**–**g**, **6** and **7** synthesized in this study are more lipophilic and less basic than the lead compound TH-701. The amidoxime **3** was directly reacted with trimethylorthoformate and boron trifluoride etherate to furnish *N*^1^,*N*^5^-bis[4-(1,2,4-oxadiazol-3-yl)phenyl]glutaramide **6** (cLog*P* and pKa values are 2.71 and −2.27 respectively). Reaction of **3** with 1,1’-carbonyldiimidazole furnished *N*^1^,*N*^5^-bis[4-(5-oxo-4,5-dihydro-1,2,4-oxadiazol-3-yl)phenyl]glutaramide **7** (cLog*P* and pKa values are 1.60 and −2.85 respectively). The procedure that we used is simple, mild and the DMSO medium can facilitate better reaction conditions due to improved solubility for a wide range of complex heteroaromatic ring systems targeted to embrace the 1,2,4-oxadiazole functionality. The trifluoro substituted 1,2,4-oxadiazole **5g** was easily formed at room temperature in 0.5 h in THF medium due to the rapid cyclization of the electron-withdrawing trifluoroacetyl ester precursor.

**Figure 4:**
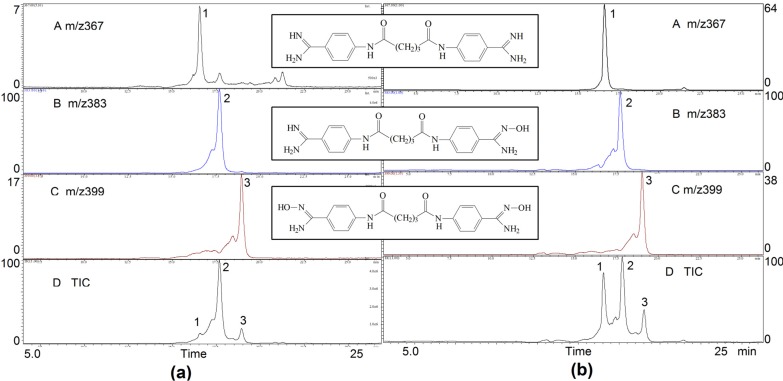
Total ion chromatograms (TIC, D) and reconstructed ion chromatograms (RIC, A, B, and C) of rat microsomal incubations collected at 60 min (**a**) and 240 min (**b**), respectively. Structures of the bis-amidoxime prodrug (C, *m/z* 399) and its two major metabolites, compound TH-701 (A, *m/z* 367) and the mono-amidoxime (B, *m/z* 383) are shown in the RICs.

Preliminary *in vitro* metabolic studies have been performed on the bis-amidoxime prodrug **3** of the lead compound TH-701. The prodrug **3** was incubated with rat liver microsomes and the metabolites were analyzed by HPLC-MS at different time intervals to obtain the chromatograms shown in Figure 4.

The two chromatograms showed in Figure 4 were obtained from incubation products collected at 60 min (a) and 240 min (b), respectively. Three major peaks have been identified as those of the prodrug **3** (peak #3, *m/z* 399), the mono-amidoxime (peak #2, *m/z* 383), and the parent bis-amidine TH-701 (peak #1, *m/z* 367). At 60 min [Figure 4(a)], most of the prodrug has been converted to the mono-amidoxime (base peak, normalized to 100), with the remaining prodrug at 17% in relative peak height, and the desired parent compound TH-701 at 7%. However, at 240 min, the peak abundance of compound TH-701 has increased significantly to 64% relative to the mono-amidoxime, and nearly twice that of the prodrug. These results indicate that the bis-amidoxime prodrug **3** of compound TH-701 undergoes facile enzymatic reduction to regenerate the desired parent compound. The mono-amidoxime intermediate is the most abundant metabolite detected in microsomal incubations, consistent with previous metabolic studies of the bis-amidoxime prodrugs for various bis-benzamidines [8].

## 3. Experimental

### 3.1. Chemistry

Unless otherwise stated, all chemicals and reagents were purchased from Sigma-Aldrich Chemical Co. (St. Louis, MO, USA) and Fisher Scientific (Waltham, MA, USA). Melting points were determined on an Electrothermal MEL-TEMP apparatus and are uncorrected. ^1^H-NMR spectra were recorded in DMSO-d_6_ on a Varian 500 MHz instrument and the chemical shift (δ) values are reported in parts per million (ppm) relative to TMS. IR spectra were recorded neat on Perkin-Elmer Spectrum 2000 instrument (samples were directly applied to the probe tip whether liquid or solid). Analytical TLC was carried out on Sigma-Aldrich (cat # Z122785-25EA), 0.2 mm percolated silica gel polyester sheets with UV indicator. Elemental analysis was carried out by M-H-W Laboratories, (Phoenix, AZ, USA). Analysis of C, H, N were within ±0.4% of theoretical values.

*N^1^,N^5^-bis[4-(N'-Hydroxycarbamimidoyl)phenyl]glutaramide* (**3**). The previously reported procedure for **3** [1] has been improved as follows. Glutaroyl dichloride (**1b**, 25 g, 18.9 mL, 148 mmol) was diluted with dimethyl formamide (25 mL). This solution was added dropwise to a solution of 4-amino-benzonitrile (**1a**, 18.4 g, 156 mmol) in dimethyl formamide (100 mL) at room temperature during 25 min. After the addition was completed the temperature of the reaction mixture was noticed to be at 55 °C. The contents were stirred at room temperature for 24 h, when a brownish white soft cake resulted. Cold 2% sodium bicarbonate (1 L) was added. The product that was separated was filtered, washed thoroughly with water, ethanol (100 mL), acetone (100 mL) and finally with hexane (50 mL). It was dried under vacuum to furnish a solid 30 g, 61% yield. The product (N^1^,N^5^-bis(4-cyanophenyl)glutaramide) (**2**) was found homogeneous on TLC (plastic back silica gel plate, mobile phase 100% ethyl acetate, R_f_ value 0.58). It was also found identical on a superimposable IR with an authentic sample earlier made at our laboratory. Compound **2** (13g, 39.1 mmol) was stirred in dimethyl sulfoxide (200 mL) at 70 °C for 20 min whereby a solution resulted. Hydroxylamine (50% solution in water, 24 mL, 391 mmol) diluted with dimethyl sulfoxide (24 mL) was slowly added to the above stirring solution at 70 °C during 15 min. The contents were stirred overnight at 70 °C for 17 h until the nitrile peak (~2214 cm^−1^) was no longer detected in the IR. The reaction mixture was cooled to room temperature and poured on to crushed ice, about 1 kg and mixed for 5 min. After the ice melted, the product that was separated was filtered, washed thoroughly with water until the washings were neutral to pH. The product was further washed in succession with cold ethanol (100 mL), cold acetone (100 mL), hexane (50 mL) and dried under vacuum to furnish a snow white solid 14.0 g, 90% yield. It was found homogeneous on TLC (plastic back silica gel plate, mobile phase dichloromethane–methanol 3:1 v/v, R_f_ 0.45) and identical with an authentic sample on a superimposable IR spectrum. This product was used for all the reactions as such in this work. Analytical sample was prepared by crystallization of the above solid from acetone-DMF-Water 8:1:1 v/v/v as shining cream white solid, mp decomposes at 225 °C; ^1^H-NMR: δ 9.99 (s, 2H), 9.50 (s, 2H), 7.59 (s, 8H), 5.71 (s, 4H), 2.39–2.37 (t, *J* = 7.5 Hz, 4H), 1.92 (m, 2H). Anal. Calcd. for C_19_H_22_N_6_O_4_ (398.42): C, 57.28; H, 5.57; N, 21.09; Found: C, 57.30; H, 5.50; N, 21.03.

#### 3.1.1. General Synthesis of Acylamidoximes **4a**–**f**

To a stirred solution of *N*^1^,*N*^5^-bis[4-(N'-hydroxycarbamimidoyl)phenyl]glutaramide (**3**, 1.0 g, 2.51 mmol) in dimethyl sulfoxide (25 mL) at room temperature was added triethylamine (0.84 mL, 6.0 mmol) and the mixture was stirred for 15 min. The appropriate anhydride (5 mmol) was then added and the contents stirred at room temperature for 18–48 h. The reaction mixture was poured on to crushed ice (500 g), stirred for 2 min and the ice was allowed to melt. The white solid separated was filtered, washed thoroughly with water and dried under vacuum.

*N^1^,N^5^-bis[4-(N'-Acetoxycarbamimidoyl)phenyl]glutaramide* (**4a**). The product was crystallized from acetone-DMF as cream white solid 0.9 g, yield 70%, mp 225–228 °C; IR: 3480 (NH_2_ stretching) 1524 (NH_2_ bending), 3336 (NH-C=O stretching), 1604 (NH-C=O bending), 1752 (ester -C=O), 1682 (NH-C=O) cm^−1^; ^1^H-NMR: δ 10.12 (s, 2H), 7.65–7.59 (m, 8H), 6.34 (s, 4H), 3.74 (s, 6H), 2.39 (t, *J* = 9.6 Hz, 4H), 2.9 (m, 2H). Anal. Calcd. for C_23_H_26_N_6_O_6_ (482.49): C, 57.25; H, 5.43; N, 17.42. Found: C, 57.16; H, 5.37; N, 17.26.

*N^1^,N^5^-bis{4-[N'-(Propionyloxy)carbamimidoyl]phenyl}glutaramide* (**4b**). The product was first crystallized from acetone-DMSO-water followed by a second crystallization from ethyl acetate-DMF as brownish white solid, 0.85 g, 66% yield and mp 186–188 °C; IR: 3486 (NH_2_ stretching), 1532 (NH_2_ bending), 3344 (NH-C=O stretching), 1620 (NH-C=O bending), 1747 (ester -C=O), 1682 (NH-C=O) cm^−1^; ^1^H-NMR: δ 10.1 (s, 2H), 7.66 (s, 8H), 6.67 (s, 4H), 3.33 (s, 6H), 2.51–2.40 (m, 12H), 1.92 (m, 2H), 1.09 (t, *J* = 8.0 Hz, 6H). Anal. Calcd. for C_25_H_30_N_6_O_6_ (510.54): C, 58.81; H, 5.92; N, 16.46. Found: C, 58.48; H, 6.00, 12; N, 16.11.

*N^1^,N^5^-bis{4-[N'-(Butyryloxy)carbamimidoyl]phenyl}glutaramide* (**4c**). The product was crystallized from acetone-DMSO 7:1 v/v as feathery white crystals 3.0 g, 60% yield, mp 194–196 °C; IR: 3484 (NH_2_ stretching), 1524 (NH_2_ bending), 3329 (NH-C=O stretching), 1614 (NH-C=O bending), 1733 (ester -C=O), 1674 (NH-C=O) cm^−1^; ^1^H-NMR: δ 10.12 (s, 2H), 7.65 (s, 8H), 6.68 (s, 4H), 2.41 (m, 6H), 1.94 (m, 2H), 1.63 (m, 4H), 0.94 (t, *J* = 9.0 Hz). Anal. Calcd. for C_26_H_34_N_6_O_6_ (526.59): C, 59.30; H, 6.51; N, 15.96. Found: C, 59.45; H, 6.36; N, 15.83.

*N^1^,N^5^-bis{4-[N'-(Hexanoyloxy)carbamimidoyl]phenyl}glutaramide* (**4d**). The product was crystallized from acetone-DMSO 5:1 v/v as a shining white solid, 1.92 g, yield 64% and mp 182–184 °C; IR: 3493 (NH_2_ stretching), 1529 (NH_2_ bending), 3345 (NH-C=O stretching), 1615 (NH-C=O bending), 1743 (ester -C=O), 1679 (NH-C=O) cm^−1^; ^1^H-NMR: δ 10.11 (s, 2H), 7.63 (s, 8H), 6.67 (s, 4H), 2.43 (m, 8H), 1.90 (m, *J* = 7.2 Hz, 2H), 1.56 (m, *J* = 7.2 Hz, 4H), 1.27 (m, 8H), 0.86 (t, *J* = 7.2 Hz, 6H). Anal. Calcd. for C_31_H_42_N_6_O_6_ (594.70): C, 62.61; H, 7.12; N, 14.13. Found: C, 62.36; H, 7.01; N, 13.96.

*N^1^,N^5^-bis{4-[N'-(Hex-5-enoyloxy)carbamimidoyl]phenyl}glutaramide* (**4e**). The product was crystallized from acetone-DMSO 9:1 v/v as a snow white solid 2.7 g, yield 73%, mp 178–180 °C; IR: 3480 (NH_2_ stretching), 1524 (NH_2_ bending), 3324 (NH-C=O stretching), 1614 (NH-C=O bending), 1733 (ester -C=O), 1674 (NH-C=O) cm^−1^; ^1^H-NMR: δ 10.12 (s, 2H), 7.65 (s, 8H), 6.70 (s, 4H), 5.87 (m, 2H), 5.11–4.99 (m, 4H), 2.55 (m, 4H), 2.41–2.35 (m, 8H), 1.92 (m, *J* = 7.2 Hz, 2H). Anal. Calcd. for C_29_H_34_N_6_O_6_ (562.62): C, 61.91; H, 6.09; N, 14.94. Found: C, 61.73; H, 5.97; N, 14.88.

*N^1^,N^5^-bis{4-[N'-(Benzoyloxy)carbamimidoyl]phenyl}glutaramide* (**4f**). The product was crystallized from acetone-DMF as snow white solid 1.1g, yield 72%, mp 253–255 °C; IR: 3507 (NH_2_ stretching), 1540 (NH_2_ bending), 3373 (NH-C=O stretching), 1643 (NH-C=O bending), 1738 (ester -C=O), 1672 (NH-C=O) cm^−1^; ^1^H-NMR: δ 10.15 (s, 2H), 8.19 (d, *J* = 7.2 Hz, 4H), 7.74–7.64 (m, 10H), 7.56–7.52 (m, 4H), 6.88 (s, 4H), 2.44 (t, *J* = 7.2 Hz, 4H), 1.94 (m, *J* = 7.2 Hz, 2H). Anal. Calcd. for C_33_H_30_N_6_O_6_ (606.63): C, 65.34; H, 4.98; N, 13.85. Found: C, 65.50; H, 4.87; N, 13.99.

#### 3.1.2. General Synthesis of 1,2,4-Oxadiazoles **5a**–**f**

N^1^,N^5^-bis[4-(N'-hydroxycarbamimidoyl)phenyl]glutaramide (**3**, 2.0 g, 5.02 mmol) was stirred in DMSO (25 mL) for 15 min at room temperature and then the appropriate anhydride (25 mmol) was added and stirred for 15 min at room temperature. The stirred solution was heated at 80–90 °C for 18–24 h and then cooled to room temperature. It was then poured on to cold (10–15 °C) 2% sodium bicarbonate solution in water (500 mL) and stirred until a solid separated. The precipitated white solid was filtered, washed thoroughly with water and dried under vacuum.

*N^1^,N^5^-bis[4-(5-Methyl-1,2,4-oxadiazol-3-yl)phenyl]glutaramide* (**5a**). The product was crystallized from methylene chloride–methanol 5:1 v/v as brownish white flakes 1.6 g, yield 69%, mp 235–237 °C; IR: 3346 (NH-C=O stretching), 1601 (NH-C=O bending), 1667 (NH-C=O) cm^−1^; ^1^H-NMR: δ 10.2 (s, 2H), 7.93 (d, *J* = 8.5 Hz, 4H), 7.79 (d, *J* = 8.5 Hz, 4H), 2.64 (s, 6H), 2.44 (t, *J* = 7.5 Hz, 4H), 1.95 (m, *J* = 7.5 Hz, 2H). Anal. Calcd. for C_23_H_22_N_6_O_4_ (446.46): C, 61.88; H, 4.97; N, 18.83. Found: C, 61.64; H, 5.18; N, 18.68.

*N^1^,N^5^-bis[4-(5-Ethyl-1,2,4-oxadiazol-3-yl)phenyl]glutaramide* (**5b**). The product was chromatographed on a column of silica gel using 100% ethyl acetate as the eluent. A white solid resulted, 1.7 g, 71% yield, mp 200–202 °C; IR: 3344 (NH-C=O stretching), 1668 (NH-C=O bending), 1692 (NH-C=O) cm^−1^; ^1^H-NMR: δ 10.22(s, 2H), 7.94 (d, *J* = 9.0 Hz, 4H), 7.79 (d, *J* = 9.0 Hz, 4H), 2.99 (quartet, *J* = 7.5 Hz, 2H), 2.44 (t, *J* = 7.5 Hz, 4H), 1.94 (m, 2H), 1.34 (t, *J* = 8 Hz, 6H). Anal. Calcd. for C_25_H_26_N_6_O_4_ (474.51): C, 63.28; H, 5.52; N, 17.71. Found: C, 63.24; H, 5.58; N, 17.86.

*N^1^,N^5^-bis[4-(5-Propyl-1,2,4-oxadiazol-3-yl)phenyl]glutaramide* (**5c**). The product was crystallized from ethyl acetate–methanol 7:1 v/v as brownish white solid 2.8 g, yield 74% and mp 196–198 °C; IR: 3323 (NH-C=O stretching), 1607 (NH-C=O bending), 1667 (NH-C=O) cm^−1^; ^1^H-NMR: δ 10.23 (s, 2H), 7.94 (d, *J* = 11 Hz, 4H), 7.80 (d, *J* = 11 Hz, 4H), 2.97 (t, *J* = 7.2 Hz, 4H), 2.46 (t, *J* = 7.2 Hz, 4H), 1.96 (m, *J* = 7.2 Hz, 2H), 1.81 (m, *J* = 7.2 Hz, 4H), 0.98 (t, *J* = 7.2 Hz, 6H). Anal. Calcd. for C_27_H_30_N_6_O_4_ (502.57): C, 64.53; H, 6.02; N, 16.72. Found: C, 64.70; H, 6.22; N, 16.87.

*N^1^,N^5^-bis[4-(5-Pentyl-1,2,4-oxadiazol-3-yl)phenyl]glutaramide* (**5d**). The product was chromatographed on a column of silica gel (3.5 × 34 cm) using ethyl acetate-hexane 1:1 and 3:1 v/v as eluents. The fractions were monitored by TLC (plastic back silica gel plate), mobile phase ethyl acetate-hexane: 19:1 v/v, R_f_ value of the product 0.74. The resulting product from the column was further crystallized from 100% methanol as shining white solid 2.6 g, yield 62%, mp 171–173 °C; IR: 3361(NH-C=O stretching), 1615 (NH-C=O bending), 1695 (NH-C=O) cm^−1^; ^1^H-NMR: δ 10.22 (s, 2H), 7.92 (d, *J* = 8.8 Hz, 4H), 7.78 (d, *J* = 8.8 Hz, 4H), 2.95 (t, *J* = 7.6 Hz, 4H), 2.44 (t, *J* = 7.2 Hz, 4H), 1.92 (m, *J* = 7.2 Hz, 2H), 1.75 (m, 4H), 1.32 (m, 8H), 0.85 (t, *J* = 7.2 Hz, 6H). Anal. Calcd. for C_31_H_38_N_6_O_4_ (558.67): C, 66.65; H, 6.86; N, 15.04. Found: C, 66.80; H, 6.65; N, 14.92.

*N^1^,N^5^-bis{4-[5-(Pent-4-en-1-yl)-1,2,4-oxadiazol-3-yl]phenyl}glutaramide* (**5e**). The product was chromatographed on a short column (3.5 × 27.5 cm) of silica gel. The product was eluted with light petroleum ether: ethyl acetate (1:3 v/v) and was further crystallized from 100% acetone as cream white rosettes, 2.1 g, yield 57%, mp 168–170 °C; IR: 3357 (NH-C=O stretching), 1601 (NH-C=O bending), 1676 (NH-C=O) cm^−1^; ^1^H-NMR: δ 10.23 (s, 2H), 7.94 (d, *J* = 8.8 Hz, 4H), 7.8 (d, *J* = 8.8 Hz), 5.93–5.83 (m, 2H), 5.14–5.01 (m, 4H), 3.09 (t, *J* = 7.6 Hz, 4H), 2.58–2.42 (m, 8H), 1.94 (m, *J* = 7.2 Hz, 2H). Anal. Calcd. for C_29_H_30_N_6_O_4_ (526.59): C, 66.15; H, 5.74; N, 15.96. Found: C, 66.28; H, 5.69; N, 15.81.

*N^1^,N^5^-bis[4-(5-Phenyl-1,2,4-oxadiazol-3-yl)phenyl]glutaramide* (**5f**). The product was chromatographed on a column of silica gel (3.5 × 45 cm) using ethyl acetate-light petroleum ether 3:1 v/v and 100% ethyl acetate where the oxadiazole was eluted. The product from the column fractions was further crystallized from acetone as cream white solid, 1.75 g, yield 61%; IR: 3313 (NH-C=O stretching), 1604 (NH-C=O bending), 1671 (NH-C=O) cm^−1^; ^1^H-NMR: δ 10.23 (s, 2H), 8.19 (d, *J* = 8.4 Hz, 4H), 8.05 (d, *J* = 8.4 Hz, 4H), 7.85 (d, *J* = 8.8 Hz, 4H), 7.76–7.64 (m, 6H), 2.46 (t, *J* = 7.2 Hz, 4H), 1.96 (m, *J* = 7.2 Hz, 2H). Anal. Calcd. for C_33_H_26_N_6_O_4_ (570.60): C, 69.46; H, 4.59; N, 14.73. Found: C, 69.31; H, 4.40; N, 14.68.

*N^1^,N^5^-bis[4-(5-Fluoro-1,2,4-oxadiazol-3-yl)phenyl]glutaramide* (**5g**). To a suspension of *N*^1^, *N*^5^-bis [4-(N'-hydroxycarbamimidoyl)phenyl]glutaramide (**3**), (1.0 g, 2.5 mmol) in anhydrous tetrahydrofuran (THF) (50 mL) was added triethylamine (2.1 mL, 99.5%, 15 mmol) at room temperature and stirred for 15 min. The reaction mixture was then cooled to 0 °C and trifluoroacetic anhydride (1.8 mL 99%, 13 mmol) diluted with THF (2.9 mL) was added during 10 min. The reaction mixture clarified into a solution after 35 min of stirring at 0–5 °C. The reaction mixture was then stirred at room temperature for 23 h. The reaction mixture was poured on to 5% sodium bicarbonate (250 mL) at 10 °C. The solid separated was filtered, washed thoroughly with water and dried under vacuum. The product was then crystallized from acetone as brownish white needles 0.91 g, yield 65%, mp 235–237 °C; IR: 3313 (NH-C=O stretching), 1615 (NH-C=O bending), 1663 (NH-C=O) cm^−1^; ^1^H-NMR: δ 10.31 (s, 2H), 8.01 (d, *J* = 9.0 Hz, 4H), 7.85 (d, *J* = 9.0 Hz, 4H), 2.48 (t, *J* = 7.5 Hz, 4H), 1.96 (m, *J* = 7.5 Hz, 2H). Anal. Calcd. for C_23_H_16_N_6_F_6_O_4_ (550.40): C, 49.83; H, 2.91; N, 15.16. Found: C, 50.04; H, 3.15; N, 15.34.

*N^1^,N^5^-bis[4-(1,2,4-Oxadiazol-3-yl)phenyl]glutaramide* (**6**). The literature procedure [10] was suitably modified as follows to prepare **6**. To a mixture of *N*^1^,*N*^5^-bis[4-(N'-hydroxy-carbamimidoyl)phenyl]glutaramide (**3**, 1.0 g, 2.5 mmol) in DMSO (25 mL) at room temperature was added trimethyl orthoformate (1.134 mL, 10.4 mmol) and stirred for 15 min when a solution resulted. Then boron trifluoride-diethyl etherate (3 drops) were added to the reaction mixture when some white cloud of fumes were seen and disappeared in 20 min. The reaction mixture was allowed to stir at room temperature for 1.0 h and then heated in an oil bath at 80–82 °C for 2 h. The reaction mixture was allowed to cool to room temperature and stirred with 150 mL of ethyl acetate. The ethyl acetate extract was successively washed with water, saturated sodium bicarbonate and water (50 mL) each. The ethyl acetate phase was concentrated to dryness under reduced pressure and crystallized from ethyl acetate-acetone-methanol 1:1 v/v as an off white granules, 0.8 g and yield 76%, mp 208–210 °C; IR: 3349 (NH-C=O stretching), 1609 (NH-C=O bending), 1706 (NH-C=O) cm^−1^; ^1^H-NMR: δ 10.30 (s, 2H), 9.65 (s, 2H), 7.99 (d, *J* = 8.4 Hz, 4H), 7.82 (d, *J* = 8.4 Hz, 4H), 2.44 (t, *J* = 7.2 Hz, 4H), 1.94 (m, *J* = 7.2 Hz, 2H). Anal. Calcd. for C_21_H_18_N_6_O_4_ (418.81): C, 60.28; H, 4.34; N, 20.09. Found: C, 60.40; H, 4.57; N, 20.21.

*N^1^,N^5^-bis{[4-(5-Oxo-4,5-dihydro)-1,2,4-oxadiazol-3-yl]phenyl}glutaramide* (**7**). The literature procedure [11] was suitably modified as follows to prepare **7**. To a solution of *N*^1^,*N*^5^-bis[4-(N'-hydroxycarbamimidoyl)phenyl]glutaramide (**3**, 1.0 g, 2.5 mmol) in DMSO (75 mL) at room temperature was added 1,1'-carbonyldiimidazole (CDI) (1.0 g, 6.2 mmol). The contents were stirred at room temperature for 30 min when a solution resulted. The reaction mixture was then heated at 100 °C for 2 h, cooled to room temperature and concentrated under high vacuum when a light brown semi-solid resulted. It was cooled in a freezer for one day when it solidified. Acetone (50 mL) was added to the product, mixed and filtered. The solid was washed with cold ethanol followed by cold acetone and hexane (50 mL) each and dried under vacuum. The product was crystallized from DMF-water as pale yellow granular solid. It was filtered, washed with water (100 mL) followed by ethanol, acetone and hexane (50 mL) each. The product was dried under vacuum to afford 0.85 g, yield 75%, mp 230 °C decomposed.; IR: 3501 (NH-C=O stretching), 1611 (NH-C=O bending), 1761 (1,2,4-oxadiazol-(4H)-5C=O), 1669 (NH-C=O) cm^−1^; ^1^H-NMR: δ 10.24 (s, 2H), 7.78–7.70 (m, 8H), 7.07 (s, 2H), 2.42 (t, *J* = 7.2 Hz, 4H), 1.91 (m, *J* = 7.2 Hz, 2H). Anal. Calcd. for C_21_H_18_N_6_O_6_ (450.41): C, 56.00; H, 4.03; N, 18.66. Found: C, 56.10; H, 4.00; N, 18.69.

### 3.2. Metabolism Studies with Prodrug **3**

The *in vitro* metabolism of the prodrug was investigated according to the method published previously [6]. In brief, the prodrug was dissolved in DMSO at a concentration of 20 mM as a stock solution of which 1 µL was added to the incubation aliquots. The 0.2-mL incubation aliquots contained 2 mg/mL rat liver microsomal proteins, 75 mM potassium phosphate (pH 7.4), 17 mM magnesium chloride, 7 mM NADPH, 17 mM glucose 6-phosphate, and 1.2 units/mL of glucose-6-phosphate dehydrogenase. Incubation times ranged from 0.5 to 4 h, each halted by placing the vials in an ice bath, followed by adding an equal volume of methanol (0.2 mL). The quenched incubation mixtures were stored at −20 °C until analysis. Before HPLC separation, microsomal proteins were precipitated by centrifugation (10,000 × *g*, 15 min) at room temperature, and the supernatant was evaporated with a stream of nitrogen at 37 °C to 0.2 mL. The residual solution was applied to a 6-mL Supelco (Bellefonte, PA, USA) C_18_ solid-phase extraction column pretreated with water and methanol. The column was washed with HPLC-grade water (2 × 3 mL) and eluted with methanol; the effluents were again concentrated by a nitrogen stream at 37 °C to 1.0 mL. Using a Zorbax **(**Agilent Technologies, Santa Clara, CA, USA) Rx-C8 column (2.1 × 150 mm; 5-µm pore size) coupled to a Supelco C_18_ guard column (2 × 18 mm, 5 µm) was used for separation. A Shimadzu LC-MS 2010 was used for initial screening of possible metabolic products generated from the microsomal incubations by obtaining the mass spectra of all chromatographic peaks. The mobile phase consisted of acetonitrile-10 mM heptansulfonate-10 mM TMAC-4.2 mM H_3_PO_4_ in H_2_O with a linear 22.5% to 45% acetonitrile gradient over 25 min.

## 4. Conclusions

A series of potential prodrugs of a promising antifungal agent, *N*^1^,*N*^5^-bis[4-(N'-(carbamimidoyl)phenyl]glutaramide, has been designed and synthesized. The highly basic and polar bis-amidine groups in the parent molecule were replaced with amidoximes, acylamidoximes or oxadiazoles functional groups that have reduced pKa values and greater partition coefficients. Metabolism study with the amidoxime prodrug showed that the designed strategy was effective in producing the bis-amidine metabolite following incubation with rat liver microsomes. Future studies will include the *in vivo* evaluation of these prodrugs in the animal model of pneumocystosis and the ability of the prodrug functions to be biotransformed into the active bis-amidine functional groups. Extension of this strategy could also be successfully applied to bis-benzamidines exhibiting potential to treat brain disorders [37,38], myotonic dystrophy [39], or Epstein-Barr virus caused diseases [40].
